# Supplementary material for: Aspartate aminotransferase-to-platelet ratio index (APRI): A potential marker for diagnosis in patients at risk of severe malaria caused by Plasmodium vivax
Source: PLoS One. 2019 Nov 25;14(11):e0224877. doi: 10.1371/journal.pone.0224877 (PMC6876935; doi:10.1371/journal.pone.0224877)
Supplement: S1 Table — (DOCX) [file pone.0224877.s001.docx]

**S1 Table.** Demographic, clinical and laboratory features of the studied patients.

| id | age | age group | sex | days_  sympt | days­­_  group | parasitemia | jaundice | dys  pnea | hem  oglob | hema  tocrit | platelet  count | plat  group | wbc | urea | ast | alt | alk  pa | tb | dir_b | ind_b | potential  _severity | apri | apri  group | hyper  alt | hyper  ast | hyper  alk_pa | hyper  tb | creat |
| --- | --- | --- | --- | --- | --- | --- | --- | --- | --- | --- | --- | --- | --- | --- | --- | --- | --- | --- | --- | --- | --- | --- | --- | --- | --- | --- | --- | --- |
| 558 | 64 | 4 | 2 | 7 | 2 | 6290 | 0 | 0 | 13 | 38.1 | 74000 | 2 | 6500 |  | 14 | 25 | 100 | 1.3 | 0.8 | 0.5 | 0 | 0.50 | 0 | 1 | 0 | 0 | 0 |  |
| 560 | 59 | 4 | 1 | 7 | 2 | 4290 | 0 | 0 | 14.1 | 41.9 | 105000 | 2 | 3900 |  | 40 | 83 | 100 | 1.1 | 0.5 | 0.6 | 0 | 1.00 | 0 | 1 | 0 | 0 | 0 |  |
| 1806 | 45 | 3 | 1 | 1 | 1 | 313 | 0 | 0 | 13.3 | 39.3 | 166000 | 3 | 5600 | 25 | 25 | 49 | 100 | 1.3 | 0.1 | 1.2 | 0 | 0.40 | 0 | 0 | 0 | 0 | 0 | 0.7 |
| 1829 | 39 | 3 | 1 | 8 | 3 | 1500 | 0 | 1 | 11.6 | 34 | 81000 | 2 | 4770 |  | 27 | 31 | 103 | 1.1 | 0.5 | 0.6 | 1 | 0.88 | 0 | 0 | 0 | 0 | 0 |  |
| 1831 | 7 | 2 | 2 | 6 | 2 | 11875 | 0 | 0 | 12.4 | 36.5 | 252000 | 3 | 6830 | 19 | 17 | 11 | 100 | 0.2 | 0.1 | 0.2 | 0 | 0.18 | 0 | 0 | 0 | 0 | 0 | 0.5 |
| 1835 | 68 | 4 | 2 | 2 | 1 | 11000 | 0 | 0 | 14.6 | 43.4 | 95000 | 2 | 8500 | 36 | 18 | 29 | 100 | 1.4 | 0.2 | 1.2 | 0 | 0.50 | 0 | 0 | 0 | 0 | 0 | 1.1 |
| 1837 | 5 | 2 | 1 | 10 | 3 | 6000 | 1 | 0 | 8 | 24.2 | 61000 | 2 | 11630 | 16 | 58 | 46 | 100 | 0.4 | 0.2 | 0.3 | 0 | 2.50 | 1 | 0 | 0 | 0 | 0 | 0.4 |
| 1838 | 16 | 2 | 1 | 1 | 1 | 2500 | 0 | 0 | 12.3 | 34.5 | 110000 | 2 | 5040 | 35 | 24 | 44 | 100 | 2.4 | 0.3 | 2.1 | 0 | 0.57 | 0 | 0 | 0 | 0 | 1 | 1 |
| 1840 | 46 | 3 | 2 | 15 | 3 | 13000 | 0 | 0 | 12.8 | 37.1 | 42000 | 1 | 2860 | 41 | 16 | 37 | 100 | 2.8 | 1.2 | 1.6 | 0 | 1.00 | 0 | 0 | 0 | 0 | 1 | 1.1 |
| 1850 | 36 | 3 | 2 | 3 | 2 | 1570 | 0 | 0 | 9.8 | 30.2 | 227000 | 3 | 5800 | 19 | 35 | 38 | 100 | 0.5 | 0.2 | 0.3 | 0 | 0.41 | 0 | 0 | 0 | 0 | 0 | 0.9 |
| 1853 | 28 | 3 | 1 | 4 | 2 | 650 | 0 | 0 | 16.2 | 47.5 | 138000 | 2 | 4980 | 23 | 27 | 22 | 100 | 0.5 | 0.1 | 0.4 | 0 | 0.51 | 0 | 0 | 0 | 0 | 0 | 1 |
| 1855 | 48 | 3 | 1 | 30 | 3 | 3670 | 0 | 0 | 8.4 | 26.5 | 194000 | 3 | 3670 | 30 | 21 | 15 | 57 | 1 | 0.3 | 0.7 | 0 | 0.28 | 0 | 0 | 0 | 0 | 0 | 1 |
| 1856 | 33 | 3 | 1 | 6 | 2 | 3000 | 0 | 0 | 12.9 | 38.3 | 73000 | 2 | 4980 | 25 | 42 | 52 | 100 | 0.9 | 0.2 | 0.7 | 0 | 1.51 | 1 | 0 | 0 | 0 | 0 | 1 |
| 1857 | 15 | 2 | 2 | 8 | 3 | 2000 | 0 | 0 | 12.7 | 37.8 | 110000 | 2 | 3720 | 17 | 81 | 136 | 215 | 0.5 | 0.1 | 0.5 | 0 | 1.94 | 1 | 1 | 1 | 1 | 0 | 0.6 |
| 1861 | 34 | 3 | 1 | 4 | 2 | 1625 | 0 | 0 | 12.5 | 37.9 | 135000 | 2 | 7100 | 29 | 18 | 43 | 134 | 0.8 | 0.4 | 0.5 | 0 | 0.35 | 0 | 0 | 0 | 0 | 0 | 0.8 |
| 1862 | 44 | 3 | 1 | 6 | 2 | 100 | 0 | 0 | 12.1 | 34.6 | 62000 | 2 | 5330 | 34 | 24 | 29 | 170 | 0.8 | 0.5 | 0.3 | 0 | 1.02 | 0 | 0 | 0 | 0 | 0 | 0.8 |
| 1863 | 17 | 2 | 1 | 8 | 3 | 1300 | 0 | 0 | 13.1 | 37.2 | 36000 | 1 | 3850 | 28 | 16 | 12 | 155 | 1.1 | 0.3 | 0.8 | 0 | 1.17 | 0 | 0 | 0 | 0 | 0 | 0.7 |
| 1864 | 40 | 3 | 1 | 13 | 3 | 5000 | 0 | 0 | 13.7 | 39.6 | 34000 | 1 | 5190 | 42 | 15 | 14 | 100 | 1 |  |  | 0 | 1.16 | 0 | 0 | 0 | 0 | 0 | 0.8 |
| 1868 | 44 | 3 | 1 | 15 | 3 | 6000 | 0 | 0 | 13.3 | 39.2 | 28000 | 1 | 2730 | 26 | 34 | 36 | 181 | 0.9 | 0.3 | 0.6 | 0 | 3.20 | 1 | 0 | 0 | 0 | 0 | 1 |
| 1872 | 48 | 3 | 2 | 10 | 3 | 163 | 0 | 0 | 11.2 | 33.8 | 208000 | 3 | 4410 | 20 | 16 | 36 | 177 | 0.4 | 0.1 | 0.3 | 0 | 0.20 | 0 | 0 | 0 | 0 | 0 | 0.7 |
| 1873 | 19 | 2 | 2 | 1 | 1 | 525 | 0 | 0 | 12.7 | 38.6 | 183000 | 3 | 4410 | 20 | 22 | 20 | 180 | 0.3 | 0.1 | 0.2 | 0 | 0.32 | 0 | 0 | 0 | 0 | 0 | 0.6 |
| 1875 | 54 | 4 | 1 | 3 | 2 | 5000 | 0 | 0 | 14.6 | 43.5 | 145000 | 2 | 3350 | 28 | 22 | 31 | 110 | 1.4 | 0.3 | 1.1 | 0 | 0.40 | 0 | 0 | 0 | 0 | 0 | 1.1 |
| 1876 | 28 | 3 | 2 | 12 | 3 | 10500 | 1 | 0 | 7.1 | 20.7 | 205000 | 3 | 7010 | 12 | 52 | 43 | 152 | 0.5 | 0.3 | 0.2 | 0 | 0.67 | 0 | 0 | 0 | 0 | 0 | 0.7 |
| 1879 | 58 | 4 | 1 | 2 | 1 | 25000 | 0 | 0 | 11.9 | 36.3 | 50000 | 1 | 5600 | 50 | 30 | 25 | 149 | 1.9 | 1.2 | 0.7 | 1 | 1.58 | 1 | 0 | 0 | 0 | 0 | 1.1 |
| 1880 | 46 | 3 | 1 | 7 | 2 | 2000 | 0 | 0 | 11.4 | 34.9 | 93000 | 2 | 4430 | 31 | 30 | 39 | 100 | 1.5 | 0.6 | 0.9 | 0 | 0.85 | 0 | 0 | 0 | 0 | 0 | 0.9 |
| 1881 | 32 | 3 | 1 | 2 | 1 | 210 | 0 | 0 | 16.5 | 48.5 | 195000 | 3 | 4480 | 25 | 30 | 24 | 117 | 0.4 | 0 | 0.4 | 0 | 0.40 | 0 | 0 | 0 | 0 | 0 | 1.1 |
| 1882 | 46 | 3 | 1 | 15 | 3 | 650 | 0 | 0 | 12 | 35.7 | 76000 | 2 | 2680 | 34 | 61 | 134 | 164 | 1.3 | 0.4 | 0.9 | 0 | 2.11 | 1 | 1 | 0 | 0 | 0 | 1 |
| 1883 | 6 | 2 | 2 | 3 | 2 | 5000 | 0 | 0 | 10.5 | 31.1 | 30000 | 1 | 8330 | 29 | 27 | 21 | 100 | 1 |  |  | 0 | 2.37 | 1 | 0 | 0 | 0 | 0 | 0.4 |
| 1891 | 51 | 4 | 1 | 9 | 3 | 4050 | 0 | 0 | 13.5 | 41 | 107000 | 2 | 5320 | 45 | 30 | 48 | 262 | 0.7 | 0.2 | 0.5 | 0 | 0.74 | 0 | 0 | 0 | 1 | 0 | 1.2 |
| 1894 | 5 | 2 | 2 | 4 | 2 | 1000 | 0 | 0 | 11.8 | 37.1 | 175000 | 3 | 3860 | 16 | 39 | 28 | 680 | 0.3 | 0 | 0.3 | 0 | 0.59 | 0 | 0 | 0 | 1 | 0 | 0.5 |
| 1895 | 43 | 3 | 1 | 3 | 2 | 20 | 0 | 0 | 12.2 | 35.7 | 145000 | 2 | 4790 | 29 | 28 | 43 | 228 | 0.4 | 0.1 | 0.3 | 0 | 0.51 | 0 | 0 | 0 | 1 | 0 | 1.1 |
| 1896 | 37 | 3 | 2 | 15 | 3 | 8000 | 0 | 0 | 10.7 | 32.1 | 185000 | 3 | 4290 | 22 | 12 | 10 | 116 | 0.5 | 0.2 | 0.3 | 0 | 0.17 | 0 | 0 | 0 | 0 | 0 | 0.7 |
| 1897 | 52 | 4 | 1 | 4 | 2 | 13500 | 0 | 1 | 12.8 | 38.9 | 50000 | 1 | 2920 | 35 | 44 | 38 | 100 | 1.9 | 1 | 1 | 1 | 2.32 | 1 | 0 | 0 | 0 | 0 | 0.9 |
| 1902 | 35 | 3 | 1 | 2 | 1 | 4500 | 0 | 0 | 13 | 38.8 | 103000 | 2 | 6660 | 20 | 25 | 37 | 100 | 1 | 0.3 | 0.7 | 0 | 0.64 | 0 | 0 | 0 | 0 | 0 | 0.7 |
| 1906 | 5 | 2 | 1 | 15 | 3 | 12000 | 0 | 0 | 2.4 | 29.1 | 55000 | 2 | 4450 | 29 | 35 | 23 | 394 | 0.6 | 0.3 | 0.3 | 1 | 1.67 | 1 | 0 | 0 | 1 | 0 | 0.5 |
| 1908 | 14 | 2 | 2 | 7 | 2 | 12000 | 0 | 0 | 12 | 35.9 | 78000 | 2 | 4660 | 27 | 19 | 13 | 100 | 2.4 | 0.7 | 1.7 | 0 | 0.64 | 0 | 0 | 0 | 0 | 1 | 0.6 |
| 1913 | 43 | 3 | 1 | 5 | 2 | 1025 | 0 | 0 | 15.3 | 44.7 | 190000 | 3 | 6170 | 13 | 26 | 34 | 112 | 0.6 | 0.2 | 0.4 | 0 | 0.36 | 0 | 0 | 0 | 0 | 0 |  |
| 1915 | 28 | 3 | 1 | 1 | 1 | 310 | 0 | 0 | 14.9 | 45.2 | 157000 | 3 | 5210 | 37 | 30 | 25 | 140 | 1 | 0.4 | 0.6 | 0 | 0.50 | 0 | 1 | 0 | 0 | 0 | 1 |
| 1918 | 36 | 3 | 1 | 5 | 2 | 4000 | 0 | 0 | 13.3 | 41.8 | 81000 | 2 | 5020 | 37 | 15 | 24 | 100 | 1 | 0.2 | 0.8 | 0 | 0.49 | 0 | 0 | 0 | 0 | 0 | 1 |
| 1919 | 36 | 3 | 1 | 4 | 2 | 4500 | 0 | 0 | 12.1 | 35.6 | 31000 | 1 | 6730 | 31 | 59 | 94 | 157 | 1.3 | 0.6 | 0.7 | 0 | 5.01 | 1 | 1 | 0 | 0 | 0 | 1.2 |
| 1922 | 51 | 4 | 1 | 9 | 3 | 4000 | 0 | 0 | 11.6 | 34.3 | 46000 | 1 | 2440 | 30 | 22 | 29 | 68 | 1.1 | 0.6 | 0.5 | 0 | 1.26 | 0 | 0 | 0 | 0 | 0 | 1.1 |
| 1930 | 49 | 3 | 2 | 60 | 3 | 3550 | 0 | 0 | 9.1 | 28.8 | 184000 | 3 | 5960 | 19 | 15 | 6 | 132 | 0.4 | 0.2 | 0.2 | 0 | 0.21 | 0 | 0 | 0 | 0 | 0 | 0.7 |
| 1931 | 57 | 4 | 1 | 29 | 3 | 5500 | 1 | 0 | 10.1 | 29.2 | 100000 | 2 | 3710 | 29 | 15 | 11 | 89 | 1.4 | 0.7 | 0.8 | 0 | 0.39 | 0 | 0 | 0 | 0 | 0 | 0.9 |
| 1938 | 32 | 3 | 2 | 3 | 2 | 6320 | 0 | 0 | 9.7 | 29.7 | 113000 | 2 | 5600 | 27 | 26 | 29 | 127 | 0.7 | 0.3 | 0.4 | 0 | 0.61 | 0 | 0 | 0 | 0 | 0 | 0.6 |
| 1942 | 26 | 3 | 2 | 5 | 2 | 2070 | 0 | 0 | 13.1 | 38.4 | 65000 | 2 | 4200 | 15 | 36 | 33 | 110 | 0.8 | 0.3 | 0.5 | 0 | 1.46 | 0 | 0 | 0 | 0 | 0 | 0.7 |
| 1944 | 47 | 3 | 1 | 3 | 2 | 22950 | 0 | 0 | 13 | 36.8 | 86000 | 2 | 4700 | 32 | 30 | 43 | 114 | 1.2 | 0.4 | 0.8 | 1 | 0.92 | 0 | 0 | 0 | 0 | 0 | 0.9 |
| 1946 | 57 | 4 | 1 | 3 | 2 | 16500 | 0 | 0 | 16.2 | 45.6 | 121000 | 2 | 9500 | 36 | 28 | 21 | 108 | 2.4 | 0.6 | 1.8 | 0 | 0.61 | 0 | 0 | 0 | 0 | 1 | 1.1 |
| 1950 | 26 | 3 | 1 | 3 | 2 | 1750 | 0 |  | 12.1 | 36.9 | 237000 | 3 | 10700 | 29 | 12 | 23 | 100 | 0.9 | 0.3 | 0.7 | 0 | 0.13 | 0 | 0 | 0 | 0 | 0 | 1 |
| 1954 | 46 | 3 | 1 | 7 | 2 | 3500 | 0 | 0 | 15.6 | 44.2 | 64000 | 2 | 5100 | 23 | 48 | 117 | 291 | 2 | 1.3 | 0.7 | 0 | 1.97 | 1 | 1 | 0 | 1 | 0 | 1.1 |
| 1957 | 24 | 3 | 1 | 4 | 2 | 3000 | 0 | 0 | 15.5 | 44.4 | 121000 | 2 | 7500 | 16 | 22 | 16 | 110 | 1.3 | 0.4 | 0.8 | 0 | 0.48 | 0 | 0 | 0 | 0 | 0 | 0.8 |
| 1962 | 40 | 3 | 1 | 5 | 2 | 4500 | 0 | 0 | 15 | 41.8 | 124000 | 2 | 9900 | 25 | 18 | 30 | 164 | 1.7 | 0.5 | 1.2 | 0 | 0.38 | 0 | 0 | 0 | 0 | 0 | 0.8 |
| 1963 | 51 | 4 | 1 | 3 | 2 | 16700 | 0 | 0 | 13.9 | 40 | 248000 | 3 | 7700 | 37 | 20 | 33 | 96 | 0.5 | 0.1 | 0.4 | 0 | 0.21 | 0 | 0 | 0 | 0 | 0 | 1.2 |
| 1965 | 23 | 3 | 1 | 7 | 2 | 22100 | 0 |  | 14.2 | 41.2 | 55000 | 2 | 6300 | 17 | 232 | 19 | 119 | 0.9 | 0.4 | 0.5 | 1 | 11.10 | 1 | 0 | 1 | 0 | 0 | 0.7 |
| 1969 | 21 | 3 | 1 | 3 | 2 | 29200 | 0 |  | 13.2 | 37 | 74000 | 2 | 8800 | 35 | 34 | 18 | 144 | 3.4 | 1 | 2.4 | 1 | 1.21 | 0 | 0 | 0 | 0 | 1 | 1 |
| 1970 | 59 | 4 | 1 | 4 | 2 | 3000 | 0 |  | 14.3 | 40.6 | 75000 | 2 | 5000 | 20 | 26 | 46 | 238 | 1.1 | 0.4 | 0.7 | 0 | 0.91 | 0 | 0 | 0 | 1 | 0 | 1 |
| 1973 | 48 | 3 | 1 | 10 | 3 | 950 | 0 | 0 | 16.9 | 48 | 142000 | 2 | 5400 | 18 | 43 | 51 | 167 | 0.8 | 0.2 | 0.6 | 0 | 0.80 | 0 | 0 | 0 | 0 | 0 | 0.9 |
| 1974 | 25 | 3 | 1 | 7 | 2 | 6500 | 0 | 0 | 15.7 | 45.2 | 170000 | 3 | 5100 | 17 | 45 | 97 | 180 | 0.9 | 0.3 | 0.6 | 0 | 0.70 | 0 | 1 | 0 | 0 | 0 | 0.9 |
| 1978 | 30 | 3 | 1 | 10 | 3 | 25 | 0 | 0 | 14.6 | 45.4 | 214000 | 3 | 4500 | 17 | 17 | 26 | 136 | 0.3 | 0.1 | 0.2 | 0 | 0.21 | 0 | 0 | 0 | 0 | 0 | 1 |
| 1980 | 24 | 3 | 1 | 8 | 3 | 9000 | 0 | 0 | 11.2 | 33.6 | 86000 | 2 | 7100 | 39 | 34 | 41 | 109 | 1.7 | 0.6 | 1.1 | 0 | 1.04 | 0 | 0 | 0 | 0 | 0 | 1.3 |
| 1981 | 33 | 3 | 1 | 8 | 3 | 7500 | 0 | 0 | 13.3 | 37.9 | 65000 | 2 | 5200 | 19 | 75 | 104 | 181 | 3.3 | 1.7 | 1.6 | 0 | 3.08 | 1 | 1 | 0 | 0 | 1 | 0.9 |
| 1982 | 47 | 3 | 1 | 7 | 2 | 5000 | 0 | 0 | 14.8 | 42.8 | 132000 | 2 | 5500 | 25 | 22 | 24 | 108 | 1 | 0.2 | 0.8 | 0 | 0.44 | 0 | 0 | 0 | 0 | 0 | 0.8 |
| 1986 | 26 | 3 | 1 | 3 | 2 | 44500 | 0 | 0 | 12.6 | 37.1 | 92000 | 2 | 2800 | 40 | 26 | 34 | 121 | 3.1 | 1.1 | 2 | 1 | 0.74 | 0 | 0 | 0 | 0 | 1 | 1 |
| 1999 | 29 | 3 | 2 | 20 | 3 | 2000 | 0 |  | 8.5 | 24.8 | 113000 | 2 | 3800 | 16 | 35 | 8 | 174 | 2.3 | 1.5 | 0.8 | 0 | 0.82 | 0 | 0 | 0 | 0 | 1 | 0.6 |
| 2001 | 60 | 4 | 1 | 2 | 1 | 21000 | 0 | 0 | 14.1 | 41.4 | 244000 | 3 | 6900 | 31 | 14 | 10 | 136 | 1.3 | 0.3 | 1 | 1 | 0.15 | 0 | 0 | 0 | 0 | 0 | 0.9 |
| 2003 | 32 | 3 | 1 | 10 | 3 | 7500 | 0 | 0 | 13.3 | 39.1 | 113000 | 2 | 4800 | 30 | 15 | 19 | 190 | 0.7 | 0.1 | 0.6 | 0 | 0.35 | 0 | 0 | 0 | 0 | 0 | 1.1 |
| 2005 | 57 | 4 | 1 | 1 | 1 | 7200 | 0 | 0 | 12.6 | 38.3 | 171000 | 3 | 8600 | 51 | 56 | 110 | 293 | 3.8 | 1.8 | 2 | 0 | 0.86 | 0 | 1 | 0 | 1 | 1 | 1.4 |
| 2007 | 34 | 3 | 2 | 7 | 2 | 3650 | 0 | 0 | 11.8 | 35.2 | 209000 | 3 | 4900 | 17 | 33 | 47 | 156 | 0.8 | 0.2 | 0.6 | 0 | 0.42 | 0 | 0 | 0 | 0 | 0 | 0.7 |
| 2013 | 38 | 3 | 1 | 7 | 2 | 15000 | 0 | 0 | 15 | 44.3 | 65000 | 2 | 5300 | 34 | 56 | 25 | 253 | 1.5 | 0.8 | 0.7 | 0 | 2.27 | 1 | 1 | 0 | 1 | 0 |  |
| 2014 | 62 | 4 | 1 | 12 | 3 | 12000 | 0 | 1 | 12.7 | 39 | 50000 | 1 | 10300 | 113 | 62 | 77 | 258 | 1.3 | 0.6 | 0.7 | 1 | 3.26 | 1 | 0 | 0 | 1 | 0 | 1.7 |
| 2015 | 43 | 3 | 1 | 8 | 3 | 8700 | 0 | 0 | 14.1 | 42 | 131000 | 2 | 9900 | 27 | 12 | 22 | 147 | 1.6 | 0.4 | 1.2 | 0 | 0.24 | 0 | 0 | 0 | 0 | 0 | 0.7 |
| 2016 | 31 | 3 | 1 | 15 | 3 | 1500 | 0 | 0 | 13 | 38.6 | 254000 | 3 | 6000 | 25 | 33 | 27 | 294 | 0.9 | 0.5 | 0.4 | 0 | 0.34 | 0 | 0 | 0 | 1 | 0 | 1.3 |
| 2017 | 38 | 3 | 2 | 8 | 3 | 1900 | 0 | 0 | 13.1 | 38.7 | 45000 | 1 | 4500 | 18 | 22 | 22 | 105 | 0.8 | 0.2 | 0.6 | 0 | 1.29 | 0 | 0 | 0 | 0 | 0 | 0.8 |
| 2020 | 56 | 4 | 1 | 2 | 1 | 450 | 0 | 1 | 15.4 | 44.6 | 90000 | 2 | 4600 | 37 | 12 | 10 | 99 | 1.2 | 0.3 | 0.9 | 1 | 0.35 | 0 | 0 | 0 | 0 | 0 | 1.1 |
| 2021 | 23 | 3 | 1 | 3 | 2 | 1900 | 0 | 0 | 13.9 | 41.4 | 82000 | 2 | 7200 | 49 | 18 | 13 | 256 | 1 | 0.3 | 0.7 | 0 | 0.58 | 0 | 0 | 0 | 1 | 0 | 1 |
| 2024 | 53 | 4 | 1 | 3 | 2 | 400 | 0 | 0 | 17.2 | 51.4 | 166000 | 3 | 4400 | 28 | 62 | 92 | 133 | 0.5 | 0.2 | 0.3 | 0 | 0.98 | 0 | 1 | 0 | 0 | 0 | 0.8 |
| 2028 | 56 | 4 | 1 | 4 | 2 | 15350 | 0 | 0 | 16.1 | 46.9 | 185000 | 3 | 8300 | 38 | 19 | 37 | 209 | 0.8 | 0.2 | 0.6 | 0 | 0.27 | 0 | 0 | 0 | 1 | 0 | 1.1 |
| 2032 | 44 | 3 | 1 | 10 | 3 | 350 | 1 | 0 | 12.5 | 36.4 | 153000 | 3 | 2700 | 28 | 16 | 11 | 137 | 0.6 | 0.1 | 0.5 | 0 | 0.28 | 0 | 0 | 0 | 0 | 0 | 0.8 |
| 2037 | 48 | 3 | 2 | 6 | 2 | 7200 | 0 | 0 | 11.1 | 34.1 | 31000 | 1 | 7000 | 48 | 34 | 45 | 467 | 2.3 | 1.6 | 0.7 | 0 | 2.89 | 1 | 0 | 0 | 1 | 1 | 1.2 |
| 2043 | 31 | 3 | 1 | 7 | 2 | 900 | 0 | 0 | 12.1 | 36.2 | 106000 | 2 | 5400 | 26 | 23 | 21 | 143 | 1.6 | 0.4 | 1.2 | 0 | 0.57 | 0 | 0 | 0 | 0 | 0 | 0.8 |
| 2045 | 39 | 3 | 1 | 7 | 2 | 550 | 0 | 0 | 8.9 | 26.9 | 175000 | 3 | 6000 | 28 | 21 | 22 | 114 | 0.5 | 0.2 | 0.3 | 0 | 0.32 | 0 | 0 | 0 | 0 | 0 | 0.8 |
| 2049 | 38 | 3 | 1 | 10 | 3 | 11400 | 1 | 0 | 12.8 | 37.9 | 61000 | 2 | 6900 | 35 | 88 | 83 | 259 | 6.4 | 4.8 | 1.5 | 0 | 3.80 | 1 | 1 | 1 | 1 | 1 | 1.2 |
| 2053 | 55 | 4 | 1 | 2 | 1 | 215 | 0 | 0 | 16.4 | 48.3 | 103000 | 2 | 5600 | 18 | 64 | 64 | 197 | 0.6 | 0.2 | 0.4 | 1 | 1.64 | 1 | 0 | 0 | 0 | 0 | 5.7 |
| 2057 | 25 | 3 | 2 | 10 | 3 | 2550 | 0 | 0 | 12.4 | 36.8 | 86000 | 2 | 4000 | 28 | 34 | 34 | 108 | 0.8 | 0.3 | 0.5 | 0 | 1.04 | 0 | 0 | 0 | 0 | 0 | 0.9 |
| 2059 | 27 | 3 | 1 | 8 | 3 | 12900 | 1 | 0 | 9.7 | 29.1 | 120000 | 2 | 6000 | 27 | 33 | 31 | 293 | 2.3 | 0.8 | 1.4 | 0 | 0.72 | 0 | 0 | 0 | 1 | 1 | 0.9 |
| 2063 | 78 | 4 | 2 | 17 | 3 | 16950 | 0 | 1 | 10.1 | 31.1 | 15000 | 1 | 6500 | 94 | 37 | 31 | 356 | 1 | 0.7 | 0.3 | 1 | 6.49 | 1 | 0 | 0 | 1 | 0 | 1.4 |
| 2064 | 69 | 4 | 1 | 2 | 1 | 14500 | 0 |  | 9 | 27.5 | 69000 | 2 | 5600 | 55 | 40 | 30 | 294 | 1.3 | 0.5 | 0.8 | 0 | 1.53 | 1 | 0 | 0 | 1 | 0 | 0.9 |
| 2067 | 35 | 3 | 1 | 3 | 2 | 11500 | 0 | 0 | 14.1 | 41.9 | 50000 | 1 | 4200 | 40 | 21 | 37 | 146 | 0.8 | 0.2 | 0.6 | 0 | 1.11 | 0 | 0 | 0 | 0 | 0 | 1.1 |
| 2069 | 59 | 4 | 1 | 3 | 2 | 1750 | 0 | 0 | 12.9 | 39 | 45000 | 1 | 4100 | 58 | 25 | 28 | 140 | 0.7 | 0.2 | 0.5 | 0 | 1.46 | 0 | 0 | 0 | 0 | 0 | 1.2 |
| 2086 | 34 | 3 | 1 | 5 | 2 | 1500 | 0 | 0 | 13.7 | 39.3 | 152000 | 3 | 4840 | 44 | 14 | 19 | 100 | 1 | 0.2 | 0.8 | 0 | 0.24 | 0 | 0 | 0 | 0 | 0 | 0.9 |
| 2087 | 56 | 4 | 1 | 3 | 2 | 5000 | 0 | 0 | 13.6 | 40.4 | 103000 | 2 | 7630 | 53 | 21 | 37 | 176 | 0.5 | 0.2 | 0.3 | 0 | 0.54 | 0 | 0 | 0 | 0 | 0 | 0.9 |
| 2091 | 59 | 4 | 1 | 3 | 2 | 6750 | 0 | 0 | 12.5 | 35.7 | 97200 | 2 | 4420 | 27 | 28 | 32 | 167 | 1.8 | 0.7 | 1.2 | 0 | 0.76 | 0 | 0 | 0 | 0 | 0 | 0.8 |
| 2109 | 37 | 3 | 1 | 3 | 2 | 1350 | 0 | 0 | 12.7 | 40 | 63000 | 2 | 4700 | 32 | 16 | 12 | 81 | 0.9 | 0.3 | 0.6 | 0 | 0.67 | 0 | 0 | 0 | 0 | 0 | 1 |
| 2110 | 37 | 3 | 1 | 9 | 3 | 11550 | 1 | 1 | 11.4 | 36.6 | 38000 | 1 | 4100 | 32 | 37 | 72 | 225 | 4.8 | 3.6 | 1.2 | 1 | 2.56 | 1 | 0 | 0 | 1 | 1 | 1.2 |
| 2112 | 34 | 3 | 2 | 5 | 2 | 22400 | 0 | 1 | 11.9 | 36 | 88000 | 2 | 4190 | 35 | 20 | 32 | 15 | 1.4 | 0.3 | 1.1 | 1 | 0.60 | 0 | 0 | 0 | 0 | 0 | 1.2 |
| 2113 | 47 | 3 | 1 | 7 | 2 | 3350 | 1 | 0 | 12.7 | 36.7 | 95000 | 2 | 6500 | 46 | 18 | 18 | 118 | 1 | 0.3 | 0.7 | 0 | 0.50 | 0 | 0 | 0 | 0 | 0 | 1.2 |
| 2117 | 63 | 4 | 1 | 3 | 2 | 400 | 0 | 0 | 16.5 | 49.8 | 77900 | 2 | 5170 | 34 | 41 | 54 | 123 | 0.9 | 0.2 | 0.7 | 0 | 1.39 | 0 | 0 | 0 | 0 | 0 | 1.2 |
| 2120 | 17 | 2 | 2 | 8 | 3 | 5850 | 0 | 0 | 11.2 | 35.5 | 70000 | 2 | 5500 | 15 | 18 | 13 | 100 | 0.6 | 0 | 0.6 | 0 | 0.68 | 0 | 0 | 0 | 0 | 0 | 0.6 |
| 2124 | 51 | 4 | 1 | 1 | 1 | 5350 | 0 | 0 | 14 | 43.3 | 160000 | 3 | 8800 | 26 | 17 | 19 | 159 | 1.2 | 0.3 | 0.9 | 0 | 0.28 | 0 | 0 | 0 | 0 | 0 | 1.1 |
| 2125 | 34 | 3 | 1 | 5 | 2 | 5000 | 0 | 0 | 12.5 | 36.7 | 137000 | 2 | 6700 | 31 | 13 | 14 | 179 | 1.1 | 0.4 | 1.2 | 0 | 0.25 | 0 | 0 | 0 | 0 | 0 | 0.9 |
| 2126 | 23 | 3 | 1 | 15 | 3 | 2500 | 0 | 0 | 15 | 43.7 | 43000 | 1 | 8600 | 29 | 23 | 29 | 139 | 1.8 | 0.4 | 1.4 | 0 | 1.41 | 0 | 0 | 0 | 0 | 0 | 1.1 |
| 2133 | 54 | 4 | 1 | 7 | 2 | 3000 | 0 | 0 | 13.1 | 38 | 66000 | 2 | 5000 | 29 | 150 | 254 | 761 | 0.6 | 0.2 | 0.4 | 0 | 5.98 | 1 | 1 | 1 | 1 | 0 | 0.8 |
| 2134 | 40 | 3 | 1 | 3 | 2 | 2500 | 0 | 0 | 15.6 | 44.7 | 109000 | 2 | 5460 | 30 | 30 | 47 | 125 | 1 | 0.3 | 0.7 | 0 | 0.72 | 0 | 0 | 0 | 0 | 0 | 1 |
| 2138 | 37 | 3 | 1 | 2 | 1 | 3500 | 0 | 0 | 12.4 | 36.6 | 125000 | 2 | 7100 | 66 | 41 | 91 | 238 | 1.9 | 0.7 | 1.2 | 0 | 0.86 | 0 | 1 | 0 | 1 | 0 | 1.1 |
| 2139 | 29 | 3 | 1 | 5 | 2 | 6500 | 0 | 0 | 15.3 | 45.1 | 22000 | 1 | 8000 | 39 | 44 | 48 | 230 | 3 | 1.2 | 1.8 | 0 | 5.26 | 1 | 0 | 0 | 1 | 1 | 1.2 |
| 2140 | 40 | 3 | 1 | 3 | 2 | 2125 | 1 | 1 | 14.2 | 42.3 | 106000 | 2 | 8600 | 27 | 14 | 16 | 97 | 5.2 | 0.5 | 4.7 | 1 | 0.35 | 0 | 0 | 0 | 0 | 1 | 0.9 |
| 2146 | 38 | 3 | 1 | 5 | 2 | 500 | 0 | 0 | 14.3 | 43.2 | 77300 | 2 | 5070 | 26 | 53 | 113 | 176 | 0.5 | 0.2 | 0.3 | 0 | 1.80 | 1 | 1 | 0 | 0 | 0 | 1.1 |
| 2147 | 56 | 4 | 2 | 11 | 3 | 2500 | 0 | 0 | 10.7 | 31.5 | 69000 | 2 | 3700 | 24 | 28 | 42 | 207 | 2.6 | 1.3 | 1.3 | 0 | 1.07 | 0 | 0 | 0 | 1 | 1 | 0.8 |
| 2150 | 45 | 3 | 1 | 2 | 1 | 250 | 0 | 0 | 14.4 | 41.5 | 147000 | 2 | 6500 | 34 | 32 | 71 | 97 | 0.5 | 0.2 | 0.3 | 0 | 0.57 | 0 | 0 | 0 | 0 | 0 | 1.2 |
| 2156 | 35 | 3 | 1 | 4 | 2 | 800 | 0 | 0 | 13.9 | 39.2 | 59000 | 2 | 3300 | 36 | 50 | 64 | 167 | 0.6 | 2 | 0.4 | 0 | 2.23 | 1 | 0 | 0 | 0 | 0 | 1.2 |
| 2158 | 5 | 2 | 2 | 7 | 2 | 2500 | 0 | 0 | 11.8 | 34 | 68000 | 2 | 3900 | 45 | 31 | 46 | 305 | 0.6 | 0.3 | 0.3 | 0 | 1.20 | 0 | 0 | 0 | 1 | 0 | 0.9 |
| 2159 | 39 | 3 | 1 | 3 | 2 | 4500 | 0 | 0 | 14.9 | 42 | 110000 | 2 | 5200 | 41 | 22 | 39 | 171 | 2.5 | 0.7 | 1.8 | 0 | 0.53 | 0 | 0 | 0 | 0 | 1 | 1 |
| 2160 | 51 | 4 | 1 | 4 | 2 | 8000 | 1 | 1 | 14.5 | 42.5 | 76000 | 2 | 5600 | 44 | 22 | 28 | 193 | 0.6 | 0.1 | 0.5 | 1 | 0.76 | 0 | 0 | 0 | 0 | 0 | 1.1 |
| 2161 | 52 | 4 | 1 | 15 | 3 | 325 | 0 | 0 | 12.3 | 35.3 | 78000 | 2 | 4400 | 24 | 14 | 25 | 152 | 0.9 | 0.3 | 0.6 | 0 | 0.47 | 0 | 0 | 0 | 0 | 0 | 0.6 |
| 2164 | 23 | 3 | 1 | 1 | 1 | 16500 | 0 |  | 13.4 | 38.2 | 114000 | 2 | 13600 | 29 | 20 | 18 | 164 | 1.4 | 0.2 | 1.2 | 0 | 0.46 | 0 | 0 | 0 | 0 | 0 | 1 |
| 2169 | 61 | 4 | 1 | 1 | 1 | 13500 | 0 | 0 | 10.9 | 32.2 | 49000 | 1 | 4100 | 33 | 36 | 46 | 344 | 1.8 | 0.7 | 1.1 | 0 | 1.93 | 1 | 0 | 0 | 1 | 0 | 0.8 |
| 2171 | 47 | 3 | 1 | 8 | 3 | 5500 | 1 | 1 | 14.2 | 41.7 | 52000 | 2 | 4100 | 28 | 63 | 149 | 411 | 1.9 | 0.8 | 1.1 | 1 | 3.19 | 1 | 1 | 0 | 1 | 0 | 1.2 |
| 2174 | 27 | 3 | 1 | 1 | 1 | 20500 | 0 | 0 | 15.3 | 47.5 | 120000 | 2 | 4500 | 23 | 15 | 17 | 134 | 1.3 | 0.3 | 0.9 | 1 | 0.33 | 0 | 0 | 0 | 0 | 0 |  |
| 2176 | 33 | 3 | 1 | 4 | 2 | 2000 | 1 | 0 | 12.9 | 37.4 | 68000 | 2 | 7800 | 31 | 27 | 64 | 100 | 5.3 | 3.4 | 1.9 | 0 | 1.04 | 0 | 0 | 0 | 0 | 1 | 1 |
| 2177 | 28 | 3 | 1 | 3 | 2 | 15500 | 0 | 0 | 11.8 | 34.9 | 18200 | 1 | 5330 | 40 | 36 | 42 | 192 | 2.5 | 1.5 | 1.1 | 0 | 5.21 | 1 | 0 | 0 | 0 | 1 | 1.5 |
| 2179 | 67 | 4 | 1 | 5 | 2 | 5000 | 0 | 0 | 12 | 35.5 | 97900 | 2 | 8090 | 34 | 15 | 18 | 138 | 0.9 | 0.3 | 0.5 | 0 | 0.40 | 0 | 0 | 0 | 0 | 0 | 0.9 |
| 2184 | 18 | 2 | 2 | 6 | 2 | 800 | 0 | 0 | 12 | 36.3 | 24000 | 1 | 5400 | 32 | 35 | 46 | 149 | 2.9 | 1.8 | 1.1 | 0 | 3.84 | 1 | 0 | 0 | 0 | 1 | 0.9 |
| 2191 | 1 | 1 | 2 | 2 | 1 | 6000 | 0 | 0 | 11.2 | 35.6 | 193000 | 3 | 12700 | 28 | 104 | 116 | 428 | 0.8 | 0.1 | 0.7 | 0 | 1.42 | 0 | 1 | 1 | 1 | 0 | 0.4 |
| 2192 | 24 | 3 | 2 | 1 | 1 | 1250 | 0 | 0 | 12.1 | 36.1 | 102000 | 2 | 6000 | 52 | 24 | 29 | 186 | 0.7 | 0 | 0.7 | 0 | 0.62 | 0 | 0 | 0 | 0 | 0 | 1.1 |
| 2194 | 48 | 3 | 1 | 5 | 2 | 600 | 0 | 0 | 10.3 | 30.6 | 72000 | 2 | 4200 | 33 | 19 | 21 | 63 | 1.5 | 0.4 | 1.1 | 0 | 0.69 | 0 | 0 | 0 | 0 | 0 | 0.8 |
| 2195 | 46 | 3 | 2 | 6 | 2 | 250 | 0 | 0 | 13 | 38.9 | 102000 | 2 | 8200 | 29 | 13 | 11 | 67 | 1 | 0.3 | 0.7 | 0 | 0.34 | 0 | 0 | 0 | 0 | 0 | 0.8 |
| 2203 | 22 | 3 | 1 | 2 | 1 | 750 | 0 | 0 | 16.2 | 44.9 | 120000 | 2 | 6200 | 25 | 19 | 18 | 100 | 1.6 | 0.3 | 1.3 | 0 | 0.42 | 0 | 0 | 0 | 0 | 0 | 1 |
| 2204 | 30 | 3 | 1 | 6 | 2 | 110 | 0 | 0 | 14.9 | 42.5 | 40000 | 1 | 6400 | 40 | 48 | 119 | 99 | 5 | 3.1 | 1.9 | 0 | 3.16 | 1 | 1 | 0 | 0 | 1 | 0.9 |
| 2207 | 39 | 3 | 1 | 3 | 2 | 400 | 0 |  | 15.3 | 43.6 | 111000 |  | 11600 | 46 | 33 | 80 | 106 | 0.7 | 0.5 | 0.3 | 0 | 0.78 | 0 | 0 | 0 | 0 | 0 | 1 |
| 2208 | 33 | 3 | 1 | 10 | 3 | 5000 | 0 |  | 13.1 | 37.2 | 62800 | 2 | 7940 | 49 | 20 | 31 | 100 | 0.7 | 0.5 | 0.1 | 0 | 0.84 | 0 | 0 | 0 | 0 | 0 | 0.9 |
| 2210 | 25 | 3 | 1 | 7 | 2 | 445 | 0 |  | 15.1 | 44.5 | 143000 | 2 | 5800 | 22 | 30 | 56 | 88 | 1.1 | 0.4 | 0.7 | 0 | 0.55 | 0 | 0 | 0 | 0 | 0 | 0.5 |

id: patient identification

age (years)

age group (1: 1-4 years; 2: 5-19 years; 3: 20-49 years; 4: ≥50 years)

sex (0: female; 1: male)

days_sympt: days from symptoms onset

days_group: days from symptom onset group (1: 1-2 days; 2: 3-7 days; 3: ≥8 days)

parasitemia: blood parasite density/mm^3^

jaundice: 0: no; 1: yes

dyspnea: 0: no; 1: yes

hemoglob: homoglobin concentration (g/dL)

hematocrit (%)

platelet count (/mm^3^)

plat group: platelet count group (1: 15,000-50,000; 2: >50,000-150,000; 3: >150,000) /mm^3^

wbc: global WBC count (/mm^3^)

urea: serum urea concentration (mg/dL)

ast: aspartate-aminotransferase (IU/L)

alt: alanine-aminotransferase (IU/L)

alk pa: alkaline phosphatase (IU/L)

tb: total bilirrubina (mg/dL)

dir_b: direct bilirrubina (mg/dL)

ind_b: indirect bilirrubina (mg/dL)

potential_severity: 0: no; 1: yes

apri:

apri group: 0: no; 1: yes

hyper alt: 0: no; 1: yes

hyper ast: 0: no; 1: yes

hyper alk_pa: 0: no; 1: yes

hyper: 0: no; 1: yes tb

creat: sérum creatinine (mg/dL)
